# Glucocorticoid use and parenteral nutrition are risk factors for catheter-related *Candida* bloodstream infection: a retrospective study

**DOI:** 10.2478/abm-2024-0016

**Published:** 2024-06-28

**Authors:** Lipeng Huang, Shanshan Li, Ronglin Jiang, Shu Lei, Jiannong Wu, Liquan Huang, Meifei Zhu

**Affiliations:** The Intensive Care Unit, Taizhou Cancer Hospital of Zhejiang Province, Taizhou, Zhejiang 317500, China; Xiaoying District Community Health Service Center, Hangzhou, Zhejiang 310006, China; The Intensive Care Unit, The First Affiliated Hospital of Zhejiang University of the Traditional Chinese Medicine, Hangzhou, Zhejiang 310006, China

**Keywords:** *Candida*, catheter-related bloodstream infection, central venous catheter, mortality, risk factor

## Abstract

**Background:**

Catheter-related candidemia (CRC) is a serious catheter-related bloodstream infection (CRBSI) caused by *Candida* spp., with higher mortality than CRBSIs caused by other organisms.

**Objective:**

To identify the risk factors for *Candida* CRBSI. The clinical characteristics of 297 patients with CRBSI in a local hospital from January 2007 to June 2015 were collected, including 33 *Candida* CRBSI and 264 non-*Candida* CRBSI.

**Method:**

The associations of *Candida* CRBSI with the clinical variables were examined using univariate and multivariate analyses.

**Results:**

Multivariate analysis showed that glucocorticoid use (odds ratio [OR] = 10.313, 95% confidence interval [CI] = 2.032–52.330, *P* = 0.005) and parenteral nutrition (OR = 5.400, 95% CI = 0.472–61.752, *P* = 0.0175) were independent risk factors for *Candida* CRBSI. The most prevalent species were *Candida tropicalis* (42.4%) and *Candida albicans* (36.36%). Of the 33 *Candida* CRBSI cases, 31 (93.93%) had indwelling central venous catheters (CVC) for ≥14 d. The mortality of *Candida* CRBSI was remarkably higher than that of bacteria CRBSI. Patients with timely catheter removal and appropriate antifungal treatment had dramatically increased 28-d survival compared with those with untimely catheter removal + inappropriate antifungal treatment (88.89% vs. 0, *P* = 0.006).

**Conclusion:**

The study identified glucocorticoid use and parenteral nutrition as independent risk factors for *Candida* CRBSI. The outcome of candidemia was associated with the duration of CVC indwelling and antifungal treatment.

The use of central venous catheters (CVC) is increasing to administer therapeutics and nutrition to critically ill patients. Catheter-related bloodstream infection (CRBSI) represents the majority of severe bloodstream infections (BSI) and exhibits a high mortality rate [1]. In the United States, 34,990 CRBSIs are diagnosed every year (3.04/10,000) [2], and the mortality rate is 2.27-fold higher than that in patients without CVC [3]. CRBSIs are nosocomial, and the primary causative agents are *Staphylococcus aureus* and *Staphylococcus epidermidis* [4].

Fungal CRBSIs are highly notorious due to the difficulty in treatment, accounting for 10% of CRBSIs [1]. The majority of the fungal CRBSIs are catheter-related candidemia (CRC), with high mortality and prolonged therapy [1, 4, 5]. Several risk factors for bacterial CRSBI have been identified, with a dearth of information about *Candida spp.*-related CRSBI (CSR-CRSBI). Nagao et al. [6] have identified (1,3)-β-d-glucan in blood as a predictor of CSR-CRSBI. Yoshino et al. [7] have reported that compared with patients with non-*Candida* CRBSI, those with *Candida* CRBSI have significantly longer durations of catheter use, more frequent pre-antibiotic treatments, and more severe clinical statuses. Thus, it is critically important to identify the patients at high risk for *Candida* infection. However, there are only a few studies on the risk factors for catheter-related fungal infections (CRFI) in China, and the sample sizes are too small to generate statistical significance and clinical relevance. Therefore, there is an urgent need to identify the risk factor for CSR-CRSBI.

In this study, we investigated the potential risk factors for CSR-CRBSI. Our data may provide new information about the surveillance, prevention, and control of CSR-CRSBI.

## Materials and methods

This study was approved by the Ethics Committee of the First Affiliated Hospital of Zhejiang University of Traditional Chinese Medicine. Written informed consent was waived due to the retrospective nature of the study.

### Patients

Patients with CRBSI were recruited from the First Affiliated Hospital of Zhejiang University of Traditional Chinese Medicine between January 2007 and June 2015. The inclusion criterion was patients with CRSBI while patients without clinical manifestation of BSI or with other suspected sources of infection were excluded.

### Definition and diagnosis

According to the Guidelines for the Prevention of Intravascular Catheter-Related Infections (USA, 2011), the CRSBI definition requires specific laboratory testing that more thoroughly identifies the catheter as the source of the BSI [8]. The criteria were that the catheter-drawn blood and/or the catheter tip and the peripheral blood were all positive for the same microorganism and that the positivity time of the catheter-drawn blood and catheter tip was earlier than that of the peripheral blood. In this study, the diagnosis of CRSBI was made following the Technical Guidelines for Prevention and Control of Catheter-related Bloodstream Infections (Trial; Release date: November 29, 2010) [9]. The positivity of CRSBI was determined during CVC insertion or within 48 h of CVC removal. A patient was considered CRSBI-positive when meeting the following criteria: (1) the same pathogen isolated from the CVC tip and the peripheral venous blood sample had >15 colony forming units (CFU) semi-quantitatively or >10^2^ CFU quantitatively, or the CFU in the intra-CVC blood was 3-fold greater than that in the peripheral blood; (2) the patient had fever (>38 °C), shivering, and/or hypotension; and (3) any other potential sources of infection were ruled out. CSR-CRBSI was diagnosed when the blood samples were *Candida*-positive, according to the Guidelines for the Diagnosis and Management of *Candida* Diseases (ESCMID, 2012) [10] and the Clinical Practice Guidelines for the Management of Candidiasis: 2016 Update by the Infectious Diseases Society of America [11]. Granulocytopenia was diagnosed when granulocytes were less than 0.5 ×10^9^/L in peripheral blood [12]. The broad-spectrum antibiotics were defined according to the Danish antibiotics categorization [13].

### Data collection

Data including gender, age, duration of hospital stay, underlying disease, CVC indwelling time and position, type of CVC, mechanical ventilation, and application of parenteral alimentation, glucocorticoids, and immunosuppressants were collected.

### Statistical analysis

Statistical analysis was conducted using SPSS 19.0 (IBM, Armonk, NY, USA). Normally distributed data were expressed as the mean ± standard deviation. Non-normal distributions and unequal variances were presented as the median (interquartile range). Continuous variables were compared using the *t* test or Mann–Whitney *U* test. Count data were expressed as the rate/percentage/composition ratio and compared using the chi-square test. Fisher's exact test was used for computing the time to negative blood culture and time to fever reduction. Risk factors for *Candida* CRBSI were identified using univariate analysis and multivariate binary logistic regression analysis. The factors with a *P* value <0.05 in the univariate analysis were included in the multivariate logistic regression analysis. A *P* value <0.05 was considered statistically significant.

## Results

### General characteristics

A total of 297 patients with CRBSI were recruited, including 33 patients with *Candida* CRBSI. The clinical characteristics of the patients are summarized in **Table 1**. Of the 33 *Candida* CRBSI cases, there were 20 (60.6%) males and 13 (39.4%) females. The mean age was 67.8 ± 21.1 years (range, 27–97 years). The major underlying diseases were renal inadequacy, cerebral infarction, malignant tumor, acute leukemia, chronic obstructive pulmonary disease, chronic nephropathy, infectious shock, hypertension, and diabetes. A total of 25 (75.8%) patients had CVC-related *Candida* infections while 8 (24.2%) had PICC-related *Candida* infections. The duration of CVC indwelling was significantly shorter than that of PICC indwelling (24.8 ± 7.6 d vs. 38.0 ± 5.7 d, *P* < 0.05).

**Table 1.** Baseline characteristics of patients with *Candida* CRBSI and non-*Candida* CRBSI

**Characteristics**	***Candida* CRBSI (*n* = 33)**	**Bacterial CRSBI (*n* = 264)**	**χ^2^/*t***	***P***	
Sex (male), n (%)	20 (60.6%)	177 (67.0%)	0.545	0.461	
Age, mean (year)	67.79 ± 21.05	69.86 ± 18.07			0.544
**Underlying diseases, n (%)**					
Cerebrovascular accidents	12 (36.4%)	131 (49.6%)	2.065	0.151	
Malignant tumors	5 (15.2%)	79 (29.9%)	3.156	0.076	
Acute leukemia	7 (22.2%)	91 (34.5%)	2.332	0.127	
Chronic obstructive pulmonary disease	3 (9.09%)	57 (21.6%)	2.121	2.121	
Chronic kidney disease	8 (24.2%)	89 (33.3%)	1.912	0.167	
Infectious shock	13 (39.4%)	135 (51.1%)	1.618	0.203	
Severe trauma	3 (9.1%)	59 (22.3%)	3.121	0.077	
Severe pancreatitis	3 (9.1%)	53 (20.1%)	2.567	0.109	
Diabetes	13 (39.4%)	143 (54.2%)	2.567	0.109	
**Catheter type, n (%)**					
Single-lumen CVC catheter	14 (42.4%)	155 (58.7%)	3.173	0.075	
Double-lumen CVC catheter	11 (33.3%)	59 (22.3%)	0.151	0.698	
Single-lumen PICC catheter	8 (24.2%)	99 (37.5%)	2.237	0.135	
**Catheter retention site n (%)**					
**CVC**	25 (75.8%)	175 (66.3%)	1.196	0.274	
Subclavian vein	8 (32.0%)	29 (16.6%)	3.454	0.063	
Internal jugular vein	11 (44.0%)	99 (56.6%)	01.397	0.237	
Femoral vein	6 (24.0%)	57 (32.6%)	0.745	0.388	
**PICC**	8 (24.2%)	89 (32.1%)	1.054	0.305	
Cubital vein	5 (62.5%)	67 (75.35%)	0.627	0.429	
Median cubital vein	3 (37.5%)	22 (24.7%)	0.627	0.429	

CRBSI, catheter-related bloodstream infections; CVC, central venous catheter, PICC, peripherally inserted central venous catheters.

### Risk factors for *Candida* CRBSI

Univariate analysis showed that *Candida* CRBSI was associated with parenteral alimentation, catheter indwelling ≥14 d, glucocorticoid use (>400 mg hydrocortisone per day), immunosuppressants/chemoradiotherapy, multiple organ dysfunction syndrome (MODS), and granulocytopenia (all *P* < 0.05) (**Table 2**). Multivariate binary logistic regression analysis identified glucocorticoid use (odds ratio [OR] = 10.313, 95% confidence interval [CI] = 2.032–52.330, *P* = 0.005) and parenteral nutrition (OR = 5.400, 95% CI = 0.472–61.752, *P* = 0.0175) were independent risk factors for CSR-CRBSI (**Table 3**).

**Table 2.** Univariate analysis

**Risk factor**	***Candida* infection (*n* = 33)**	**Bacterial infection (*n* = 264)**	***P***
Mechanical ventilation (n, %)	12 (36.4%)	104 (39.4%)	0.737
Hospital stay >15 d (n, %)	28 (84.8%)	204 (77.3%)	0.321
Broad-spectrum antibiotics (n, %)	31 (93.9%)	232 (87.9%)	0.396^*^
Parenteral nutrition (n, %)	30 (90.9%)	104 (39.4%)	< 0.001
Age >65 years (n, %)	28 (84.8%)	208 (78.8%)	0.416
Diabetes or hyperglycemia (n, %)	18 (54.5%)	113 (42.8%)	0.200
>4 underlying diseases (n, %)	28 (84.8%)	195 (73.9%)	0.169
Catheter indwelling ≥14 d (n, %)	31 (93.9%)	185 (70.1%)	0.004
Renal inadequacy (n, %)	15 (45.5%)	127 (48.1%)	0.774
MODS (n, %)	14 (42.4%)	58 (22.0%)	0.010
Glucocorticoids (>400 mg hydrocortisone/d; n, %)	28 (84.8%)	74 (28.0%)	< 0.001
Immunosuppressant/chemoradiotherapy (n, %)	12 (36.4%)	37 (14.0%)	0.001
Granulocytopenia (n, %)	8 (24.2%)	16 (6.1%)	0.002^*^
Major surgery (n, %)	3 (9.1%)	16 (6.1%)	0.454^*^
Urinary catheterization (n, %)	19 (57.6%)	125 (47.3%)	0.268
Blood or albumin transfusion (n, %)	32 (97.0%)	228 (86.4%)	0.096^*^

* Fisher's exact rate method; others, chi-square test.

CRBSI, catheter-related bloodstream infections, MODS, multiple organ dysfunction syndrome.

**Table 2.** Multivariate analysis

**Risk factor**	**OR**	**95% CI**	***P***
Parenteral nutrition	5.400	0.472–61.752	0.0175
Time of catheter indwelling ≥14 d	0.488	0.043–5.486	0.561
Immunosuppressant/chemoradiotherapy	0.800	0.188–3.401	0.763
MODS	0.346	0.100–1.198	0.094
Glucocorticoids	10.313	2.032–52.330	0.005
Granulocytopenia	2.625	0.670–10.280	0.166

CI, confidence interval; CRBSI, catheter-related bloodstream infections, d, days, MODS, multiple organ dysfunction syndrome, OR, odds ratio.

### Timely catheter removal and appropriate antifungal treatment improve the outcomes of patients with *Candida* CRBSI.

By comparing the mortality between patients with *Candida* CRBSI and those with bacteria CRBSI, we found that the mortality of *Candida* CRBSI was remarkably higher than that of bacteria CRBSI (51.52% vs. 21.6%, ***χ***^2^ = 11.791, *P* = 0.006; **Table 4**). To investigate the association of timely catheter removal and appropriate antifungal treatment with the outcomes of patients with *Candida* CRBSI, we divided the 33 patients into 4 groups: timely catheter removal + appropriate antifungal treatment (n = 9), timely catheter removal + inappropriate antifungal treatment (n = 7), delayed catheter removal + appropriate antifungal treatment (n = 11), and delayed catheter removal + inappropriate antifungal treatment (n = 6). The 28-d survival rates of the 4 groups, representing the proportion of patients who survived the 28-d follow-up period, were 88.89%, 42.86%, 45.45%, and 0%, respectively (***χ***^2^ = 11.791, *P* = 0.006; **Table 5**), suggesting that both timely catheter removal and appropriate antifungal treatment are important for good outcomes of the patients.

**Table 4.** Comparison of mortality between *Candida* CRBSI and Bacterial CRBSI

	***Candida* CRBSI (*n* = 33)**	**Bacterial CRBSI (*n* = 264)**	***χ* ^2^**	***P***
Number of deaths (rates, %)	17 (51.52)	57 (21.60)	14.041	0.001

**Table 5.** Overall survival of patients with *Candida* CRBSI

**Group**	**Number of survival (rates, %)**	**Number of deaths (rates, %)**	**Survivor (n = 16)**	
			**Fever reduction time (d)**	**Time for blood culture to turn negative (d)**
Timely catheter removal + appropriate antifungal treatment (n = 9)	8 (88.89)	1 (11.11)	5.50 ± 2.27	4.38 ± 1.60
Timely catheter removal + inappropriate antifungal treatment (n = 7)	3 (42.86)	4 (57.14)	11.33 ± 1.53^*^	10.33 ± 2.52^#^
Delayed catheter removal + appropriate antifungal treatment (n = 11)	5 (45.45)	6 (54.55)	9.60 ± 2.41^*^	8.80 ± 2.28^#^
Delayed catheter removal + inappropriate antifungal treatment (n = 6)	0	6 (100.00)		
Total numbers (n = 33)	16 (48.48)	17 (51.52)	7.88 ± 3.26	6.88 ± 3.22

Overall survival was compared using Fisher's exact rate method, *P* = 0.006.

* *P* < 0.01 vs./Timely catheter removal + appropriate antifungal treatment group.

# *P* < 0.01 vs. Timely catheter removal + appropriate antifungal group.

CRBSI, catheter-related bloodstream infections.

## Discussion

Many risk factors contribute to CRBSI. *Candida* and non-*Candida* CRBSI share some common risk factors, such as antimicrobial drug use, the length of ICU stay, and hemodialysis. In this study, we demonstrated for the first time that the use of glucocorticoids and parenteral nutrition were independent risk factors for CSR-CRBSI.

The prime reason for the development of CSR-CRBSI is a decrease in the immune response, particularly in elderly patients with diabetes or hyperglycemia receiving treatments with immunosuppressants, glucocorticoids, or long-term antibiotics. Fardet et al. [14] have reported that the relative risk of candidiasis is very high during the first weeks of glucocorticoid exposure, and the risk of infection increases with age, the presence of diabetes, and the dosage of glucocorticoids. *Candida* can quickly colonize the surfaces of catheters and accelerate the formation of biofilms [15]. Lipid emulsions can promote the formation of *E. coli–C. albicans* mixed-species biofilms, increasing the risk of CRBSI [16]. In the present study, 28 patients (84.9%) used glucocorticoids that may impair the immune system, thereby reducing the phagocytic function and facilitating fungal infection. In addition, all parenteral nutrition contained lipid emulsion. The multivariate analysis showed that the use of glucocorticoids and parenteral nutrition were independent risk factors for CSR-CRBSI. In this study, 30 (90.9%) patients with *Candida* infection received parenteral nutrition. Therefore, in clinical practice, special attention is required in patients receiving glucocorticoids treatment and parenteral nutrition.

Previous reports have identified femoral vein catheterization as an independent risk factor for CSR-CRBSI [17], [18], [19], [20]. Catheter indwelling causes skin barrier damage, permitting entry of the fungi in the catheter via a subcutaneous tunnel. Nosocomial spread of the fungi can occur through intravenous drug and fluid administration [2]. Both present and previous studies suggest that parenteral nutrition is a risk factor for CSR-CRBSI [21, 22]. Therefore, reducing the inappropriate use of parenteral nutrition may prevent CSR-CRBSI. Surgery can also damage the skin barrier. In the present study, 3 patients had a history of major abdominal surgery, which is an independent risk factor for CRFI [23, 24].

Our data showed that CSR-CRBSI primarily occurred in ICU and the Department of Hematology (63.6%), possibly due to increased use of central catheters, decreased immunity of patients, and prolonged use of antibiotics and chemotherapeutics in the two departments. At our hospital, both CVC and PICC are used, according to the patients' condition. The indwelling time of PICC was significantly longer than that of CVC, but the incidence of CSR-CRBSI in PICC users was lower than that in CVC users. The possible reason is that PICC is inserted via superficial veins, making the deep veins inaccessible to the pathogen due to the long travel distance [25]. Femoral vein catheterization increases the risk for bacterial contamination and has been identified as a risk factor for CRFI [17], [18], [19], [20]. The most commonly used vein for PICC is the basilic vein with a large diameter, fixed location, straight channel, relatively low number of valves, and ease of catheterization. The median cubital vein is located in the cubital fossa, and the site of catheterization can be obstructed by elbow flexion. In addition, the number of valves and the inter-individual differences are relatively high, making catheterization more difficult. Nevertheless, in this study, the infection rates between the patients with PICC in the basilica vein and those with PICC in the median cubital vein were comparable. For CVC, the indwelling time of the double-lumen catheter was significantly smaller than that of the single-lumen catheter (*P* < 0.05), possibly due to the high contact area in the double-lumen catheter. Several studies have shown that the risk for infection increases along with the increasing number of CVC lumens [26, 27]. In addition, 93.94% of patients had ≥ 14 d of catheter indwelling time. Therefore, clinically, the subclavian and basilic veins, single-lumen catheter, and the shortest possible time should be considered for minimizing the risk for CSR-CRBSI. Indeed, the IDSA guidelines recommend the subclavian vein for catheter indwelling, followed by internal jugular and femoral veins [9].

In this study, the most common *Candida spp.* isolated from CSR-CRBSI patients were *C. tropicalis* (42.4%) and *C. albicans* (36.4%). The frequencies of *C. glabrata*, *C. famata*, and *C. lusitaniae* were relatively low. The guidelines for the Treatment of Fungal Infections in Adult Pulmonary and Critical Care Patients issued by the American Thoracic Society [28] designates *C. albicans* as the most common causative agent of candidemia.

Our findings showed that the 28-d survival rates of CSR-CRBSI patients with timely catheter removal and/or appropriate antifungal treatment ranged from 45.45% to 88.89%. It has been reported that the mortality rates of candidemia patients range between 32% and 50% [29], [30], [31], supporting our findings. The *Candida* biofilms formed on the catheters' surface are difficult to treat and require 100–1,000-fold higher doses of antifungal agents, resulting in high mortality [32]. To address this issue, patients with long-term catheter indwelling should be closely monitored, blood samples should be obtained immediately for bacterial or fungal culture upon the suspicious presentations of CRI, and the CVC should be timely removed. For patients who have already been treated with high-grade sensitive antibiotics but with persisting mild or moderate inflammatory responses and coagulation disorders or abnormal bleeding, antifungal treatment should be performed at the earliest event even when positive results are still pending.

The present study has some limitations: (1) this was a single-center study, and the sample size was relatively small; (2) only the existing data were analyzed due to the retrospective nature of this study; (3) there was a lack of confounding factor collection. Some risk factors such as different setting, procedure, and sample size were not examined in this study due to lack of data; (4) the incidence of CRBSI compared with overall use of CVC was not examined; and (5) we excluded patients without clinical manifestation of BSI or with other suspected sources of infection. However, some BSI might not have obvious clinical symptoms but were still BSI. Other metrics should be considered for patient enrollment.

In conclusion, glucocorticoid use and parenteral nutrition are associated with the occurrence of CSR-CRBSI. The most common causal pathogens of CSR-CRBSI were *C. tropicalis* and *C. albicans*.
